# Biodistribution and radiation dosimetry of [^99m^Tc]Tc-N4-BTG in patients with biochemical recurrence of prostate cancer

**DOI:** 10.1186/s13550-024-01105-6

**Published:** 2024-04-26

**Authors:** Andreas Rinscheid, Alexander Gäble, Georgine Wienand, Alexander Dierks, Malte Kircher, Thomas Günther, Marianne Patt, Ralph A. Bundschuh, Constantin Lapa, Christian H. Pfob

**Affiliations:** 1 Medical Physics and Radiation Protection, University Hospital Augsburg, Augsburg, Germany; 2 Nuclear Medicine, Faculty of Medicine, University of Augsburg, Augsburg, Germany; 3 Molecular Imaging Program at Stanford, Department of Radiology, Stanford University, Palo Alto, CA USA; 4 Bavarian Cancer Research Center (BZKF), Erlangen, Bavaria Germany

**Keywords:** Prostate cancer, Theranostics, Bombesin, PET, GRPR

## Abstract

**Background:**

In patients with prostate cancer (PCa), imaging with gastrin-releasing peptide receptor (GRPR) ligands is an alternative to PSMA-targeted tracers, particularly if PSMA expression is low or absent. [^99m^Tc]Tc-N4-BTG is a newly developed GRPR-directed probe for conventional scintigraphy and single photon emission computed tomography (SPECT) imaging. The current study aims to investigate the safety, biodistribution and dosimetry of [^99m^Tc]Tc-N4-BTG in patients with biochemical recurrence (BCR) of PCa.

**Results:**

No adverse pharmacologic effects were observed. Injection of [^99m^Tc]Tc-N4-BTG resulted in an effective dose of 0.0027 ± 0.0002 mSv/MBq. The urinary bladder was the critical organ with the highest mean absorbed dose of 0.028 ± 0.001 mGy/MBq, followed by the pancreas with 0.0043 ± 0.0015 mGy/MBq, osteogenic cells with 0.0039 ± 0.0005 mGy/MBq, the kidneys with 0.0034 ± 0.0003 mGy/MBq, and the liver with 0.0019 ± 0.0004 mGy/MBq, respectively. No focal tracer uptake suggestive of PCa recurrence could be revealed for any of the patients.

**Conclusion:**

[^99m^Tc]Tc-N4-BTG appears to be a safe diagnostic agent. Compared to GRPR-targeted PET tracers, this ^99m^Tc-labelled SPECT agent could contribute to a broader application and better availability of this novel approach. Further research to assess its clinical value is warranted.

## Background

Prostate cancer (PCa) is the most common solid malignancy in men, with an incidence of 214 diagnosed cases per 1000 men [1]. Recently, positron emission tomography (PET) using ligands addressing the prostate-specific membrane antigen (PSMA) has attracted the attention of clinicians and imaging specialists and has become the new gold standard for staging of high risk PCa and localising BCR [2, 3]. Even for conventional scintigraphy and SPECT imaging several PSMA ligands are available and have shown comparable results to PET-ligands though suffering from lower sensitivity [4]. However, PSMA expression has only been observed in approximately 90–95% of PCa patients. Thus, alternatives are required [5]. Another promising molecular target for the detection of PCa is the gastrin-releasing peptide receptor (GRPR), a 7-trans-membrane G-protein-coupled receptor activating phospholipase C-signalling, which has been shown to be overexpressed especially in breast and prostate cancer [6–8]. In a large study of *n* = 530 pathological samples, sufficient GRPR expression could be proven in 77% of primary PCa [9]. Noteworthy, receptor expression seems to be inversely correlated with Gleason score [9], which might render it complementary to PSMA. Regarding clinical data, most experience has been gained with the PET agent [^68^Ga]Ga-RM2 including head-to-head comparisons to PSMA-PET [10, 11] or approaches to use its ^177^Lu-labelled analog for radioligand therapy [12]. Previously developed bombesin analogs linked to ^99m^Tc showed limited detection rates for bone metastasis and could not detect lymph node metastasis [13, 14].

Given the continuously expanding field of clinical indications for non-invasive in vivo PCa theranostics (even beyond PSMA), development of a ligand amenable to radiolabelling with gamma emitters (such as technetium-99 m) for use in conventional scintigraphy or single-photon emission computed tomography (SPECT) with its lower cost and generally better availability would be highly desirable.

**Fig. 1:**
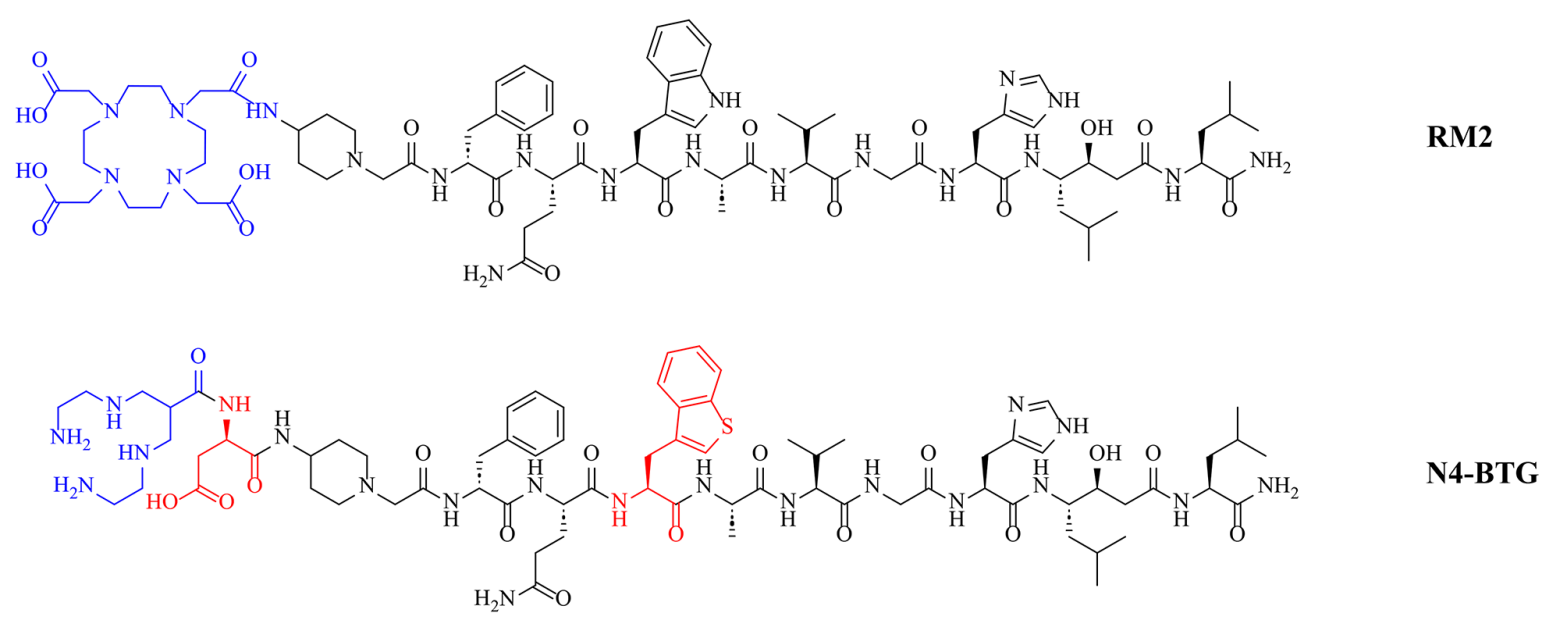
Chemical structure of RM2 and N4-BTG. Structural differences are depicted in red. Chelators (DOTA in RM2, N4 in N4-BTG) are depicted in blue

This need has been recognized, and has fuelled the recent development and preclinical characterization of ^99m^Tc-labeled tracers, such as [^99m^Tc]Tc-N4-BTG (Fig. 1), a novel RM2 analog modified in the pharmacophore to improve activity clearance and thus, tumour-to-organ ratio at early time points [15].

In this study, we present the safety, biodistribution and dosimetry for the first four patients examined with [^99m^Tc]Tc-N4-BTG, a novel ^99m^Tc-labeled GRPR antagonist. This new probe allows conventional scintigraphy and SPECT imaging addressing GRPR as the target.

## Methods

All reported investigations were conducted in accordance with the Helsinki Declaration and with national regulations. The local ethics committee (ethics committee of the Ludwig-Maximilians-Universität München, Munich, Germany) approved this retrospective analysis (permit number 22–0691). [^99m^Tc]Tc-N4-BTG was prepared in compliance with the German Medicines Act, AMG § 13 2b, and after notifying the responsible regulatory authority. All participants provided written informed consent for the imaging procedure and publication of the images.

### Patient selection

This retrospective analysis includes four consecutive male patients (Table 1) with a mean age of 66 ± 5 years (range, 58 to 70 years) showing biochemical recurrence (BCR) of prostate cancer (PCa) following radical prostatectomy. The mean observed PSA level was 1.7 ± 0.6 ng/mL (range, 1.0 to 2.4 ng/mL). Previously performed [^68^Ga]Ga-PSMA-I&T PET/CT scans had shown no evidence of local recurrence or metastases, so compassionate use [^99m^Tc]Tc-N4-BTG-SPECT/CT scans were performed. Individual patient characteristics can be found in Table 1. Safety was assessed by monitoring adverse events up to 5 h after administration of [^99m^Tc]Tc-N4-BTG.

**Table 1:** Individual patient characteristics and injected activity of [^99m^Tc]Tc-N4-BTG.

	Patient 1	Patient 2	Patient 3	Patient 4	Mean	SD
Age [y]	70	58	66	68	66	4.6
Weight [kg]	84	102	85	80	88	8
Height [cm]	170	186	172	172	175	6.4
Injected activity [MBq]	683	739	836	828	772	74
Specific activity [MBq/kg]	8.2	7.5	10.1	10.4	9.0	1.2
PSA [ng/ml]	1.2	2.1	2.4	1.0	1.7	0.6
Gleason Score	3 + 4	9	4 + 3	4 + 3		
Initial Stage	pT2c pN0 R1	pT3b pN1 R0	pT2c pN1 R1	pT2c pN0 R0		
Previous treatments	RP, RTx, ADT	RP, RTx, ADT	RP, ADT	RP, RTx		

ADT: androgen deprivation therapy, RP: radical prostatectomy, RTx: radiation therapy

### Preparation of [^99m^Tc]Tc-N4-BTG

[^99m^Tc]Tc-N4-BTG was prepared by adding up to 0.8 ml Na[^99m^Tc]TcO_4_ to a mixture of 7.5 nmol N4-BTG peptide, 5 µl SnCl_2_ solution (1 mg/ml) in sodium ascorbate (3 g/l in 0.01 M HCl), 3 µl 0.1 M disodium hydrogen citrate sesquihydrate in water, and 25 µl 0.5 M Na_2_HPO_4_ buffer (pH 9.5). The solution was heated to 98 °C for 10 min, and after cooling for 5 min it was diluted with isotonic saline to a final volume of 10–20 ml. Finally, it was sterile filtered by mean of two 0.22 μm filters in series (Cathivex and Millex GV, Millipore). Chemical and radiochemical purity was determined by means of high-performance liquid chromatography (HPLC; column: Hypersil GOLD 150 × 3; gradient: 15/85 to 95/5 (v/v) CH_3_CN/H_2_O with 0.1% TFA within 8 min; flow: 0.8 ml/min; UV (λ = 220 nm) and radiodetection; t_R_ about 5 min) and thin layer chromatography (silica gel–impregnated glass fibre sheets (Varian) and methyletherketone as eluent; Rf of [^99m^Tc]Tc-N4-BTG: 0.2). Radiochemical purity was greater than 90% and all other quality parameters (pH, endotoxin level, sterility) were within limits prescribed by the general monograph (Ph. Eur.) for radiopharmaceutical preparations. The radiopharmaceutical was administered via an intravenous bolus injection, followed by a saline flush.

### Imaging protocol

**Fig. 2:**
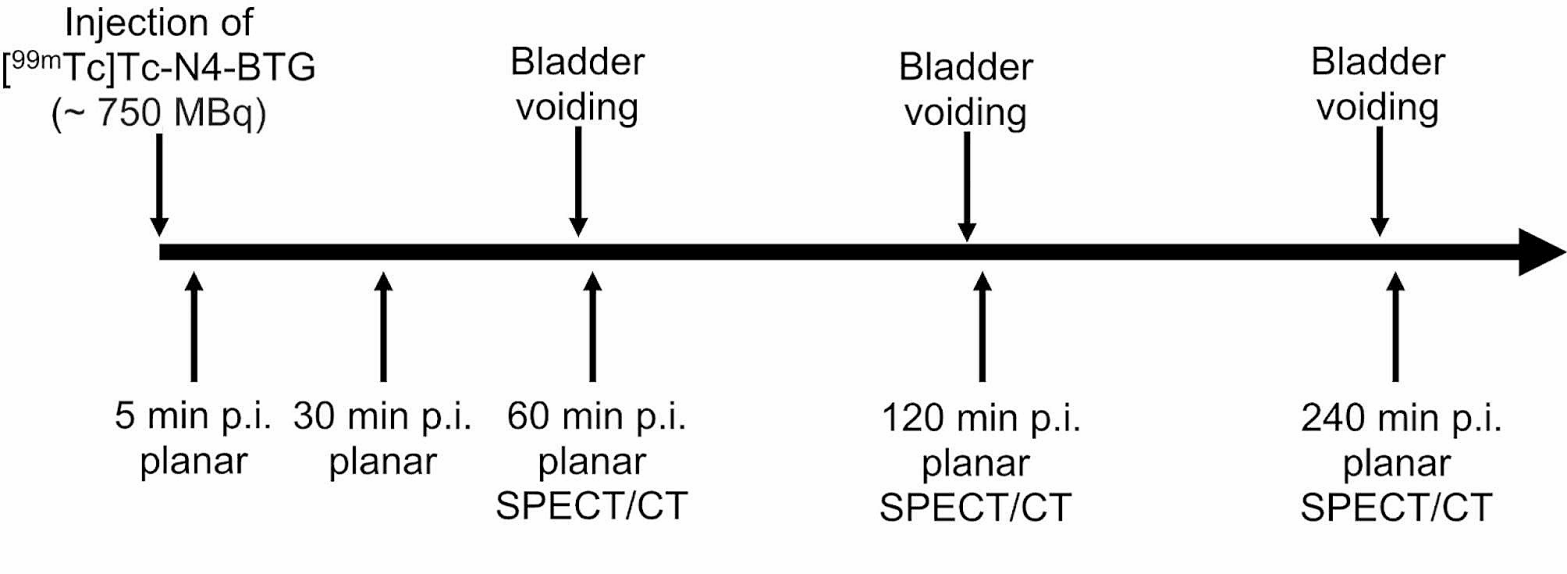
Imaging protocol for [^99m^Tc]Tc-N4-BTG.

All scans were obtained on a GE Discovery NM/CT 670 Pro (GE Healthcare, Milwaukee, USA) SPECT/CT system, equipped with a LEHR collimator and operating within an energy window of 140.5 keV ± 10%. A scatter window ranging from 114 to 126 keV was additionally acquired for planar and SPECT imaging. For SPECT, the imaging field of view was from neck to upper thighs. At each bed position, 60 views were acquired, with a duration of 8 s each. Subsequently, images were reconstructed using an ordered subset expectation maximisation (OSEM) algorithm (2 iterations and 10 subsets) with a Butterworth filter (*f*_c_ = 0.48, *n* = 10). Scatter and attenuation correction was applied. For whole-body planar imaging, a scan speed of 30 cm/min was employed until 60 min post-injection, after which the acquisition speed was moderated to 12 cm/min for any further data collection. Planar imaging was performed at 5 and 30 min after injection of the radiopharmaceutical. At 60, 120, and 240 min after injection additional SPECT/CT and planar imaging was acquired. An overview of the imaging protocol can be found in Fig. 2. For the scans at 5 and 30 min after injection, no micturition took place. Subsequently, the patients were asked to void their bladder directly prior to each examination. No blood samples were taken.

### Image analysis and dosimetry

First, a systematic visual analysis was performed by two experienced nuclear medicine physicians (AG, CHP). Potential discrepancies were resolved by consensus. The process included assessing presence of focal tracer accumulation that surpassed background activity at potential sites of tumour lesions.

For dosimetry, organ volumes were determined from the CT dataset of the corresponding SPECT/CT. Time-activity data, derived from the geometric mean of anterior and posterior planar images to compensate in part for attenuation, were assessed using the Xeleris Dosimetry Toolkit (GE Healthcare, Milwaukee, WI, USA). The first planar image (captured prior to micturition) was utilised to establish the calibration factor. Regions of interest (ROI) were manually delineated on the entire body and the urinary bladder to also estimate the whole-body clearance. Background ROIs were delineated outside the body. Time-activity data based on SPECT/CT were also assessed using the Xeleris Dosimetry Toolkit, with standard activity within the field of view used to ascertain the calibration factor. Volumes of interest (VOIs) were drawn on the CT and then transferred to the SPECT for the pancreas, kidneys, liver, spleen, heart, lungs and lumbar vertebrae 2–4. Additionally, a sphere of about 100 mL was drawn within the region of the right shoulder (mostly covering M. subscapularis) to account for background. For determining the red bone marrow (RBM) source activity, it was assumed that 6.7% of the RBM activity is within lumbar vertebrae 2–4 [16, 17]. Time-integrated activity coefficients (TIACs) were computed fitting mono-exponential functions to the data using the NUKFIT software [18, 19]. The TIAC of the urinary bladder was estimated using the whole-body clearance rate and the voiding interval of 1 h, 2 h, 4 h and every 3.5 h from then on. Individual patient absorbed doses for the whole body and organs of interest were estimated based on the MIRD schemes using OLINDA/EXM version 1.0 [20] taking into account the patients‘ individual organ masses. The effective dose provided by OLINDA/EXM was corrected using the current tissue-weighting factors from ICRP 103 [21].

### Statistics

All continuous data are reported as mean, standard deviation and range.

## Results

### Radioligand and patient safety

The administered mass of [^99m^Tc]Tc-N4-BTG was 12 ± 1 µg. The overall injected activity (radiochemical purity greater than 98%) ranged from 683 to 836 MBq (mean ± SD, 772 ± 74 MBq). Injection of [^99m^Tc]Tc-N4-BTG was well tolerated by all four subjects. No side effects or changes in vital signs were observed during the study or follow-up period.

### Image analysis

**Fig. 3:**
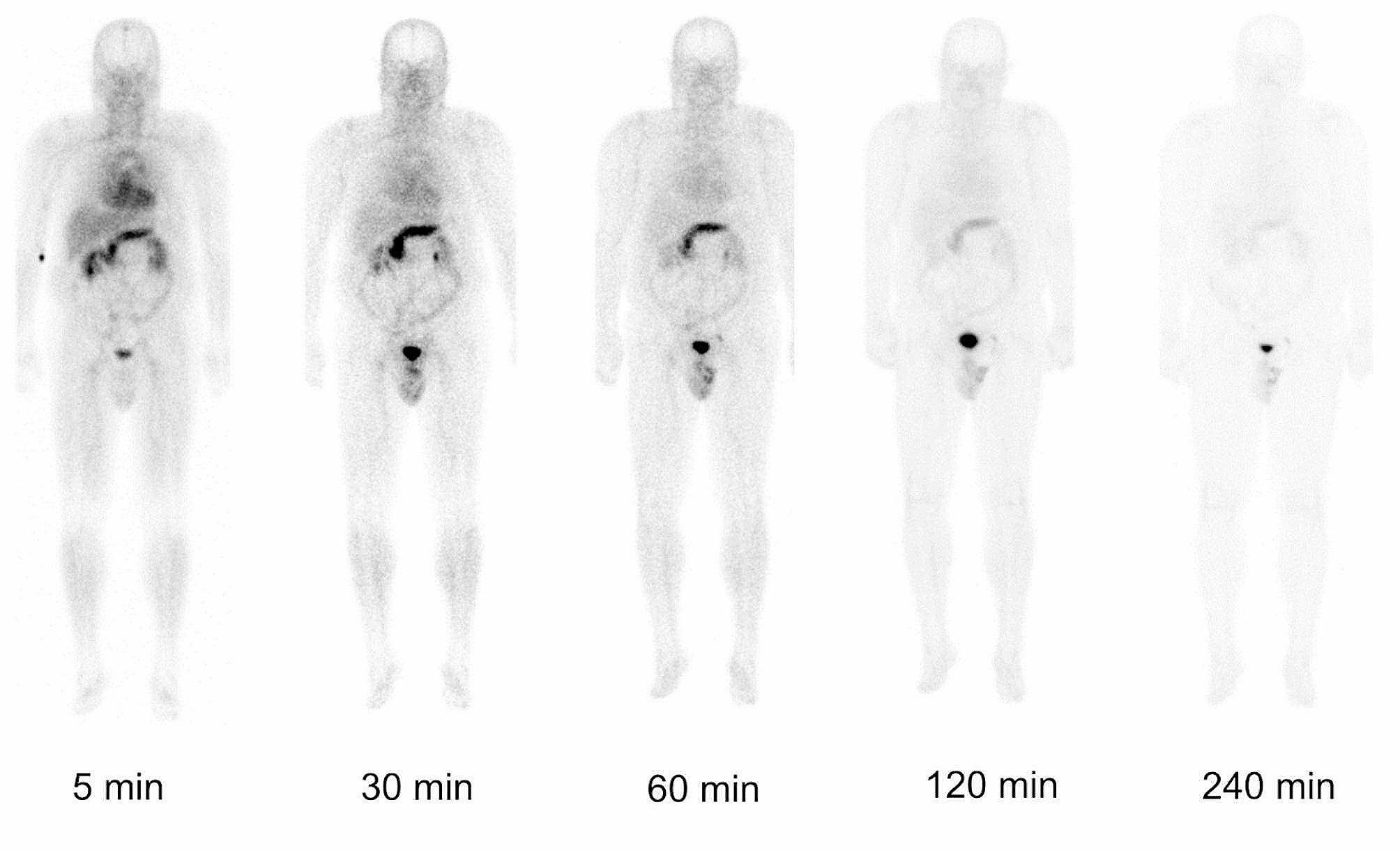
Sequential anterior views of whole-body planar scans of a 70-y-old patient (P1; PSA level 1.2 ng/ml). Biodistribution of ^[99m^Tc]Tc-N4-BTG is depcited at 5, 30, 60, 120 and 240 min after i.v. administration

Visual image analysis showed swift activity clearance from blood/background via rapid renal excretion. High physiological uptake of [^99m^Tc]Tc-N4-BTG was seen in pancreas, liver and spleen immediately after injection, whereas no focal lesions suggestive for PCa manifestations could be recorded at any measurement. The pancreas exhibited maximum uptake only after 30 min, followed by a fast washout phase. Most of the activity accumulated in liver and spleen at 5 min after injection, and was cleared within the first 30 min after injection. Whole-body planar images at different time points for Patient 1 (P1) are displayed in Fig. 3. Patient 3 (P3) exhibited high tracer retention in the lymphatic tissue of the Waldeyer’s ring, consistent with inflammatory changes.

### Biodistribution

**Fig. 4:**
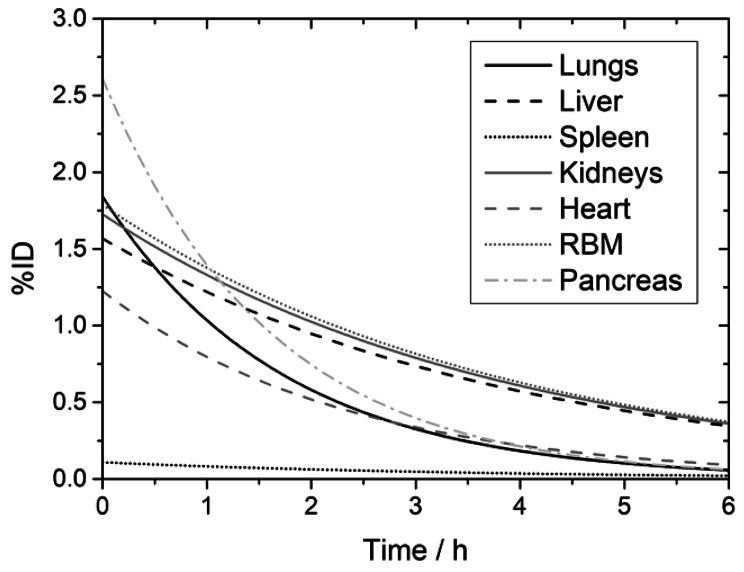
Time-activity curves for Patient 1 (P1) for all delineated organs and the red bone marrow (RBM) showing percentage of injected dose (%ID).

**Fig. 5:**
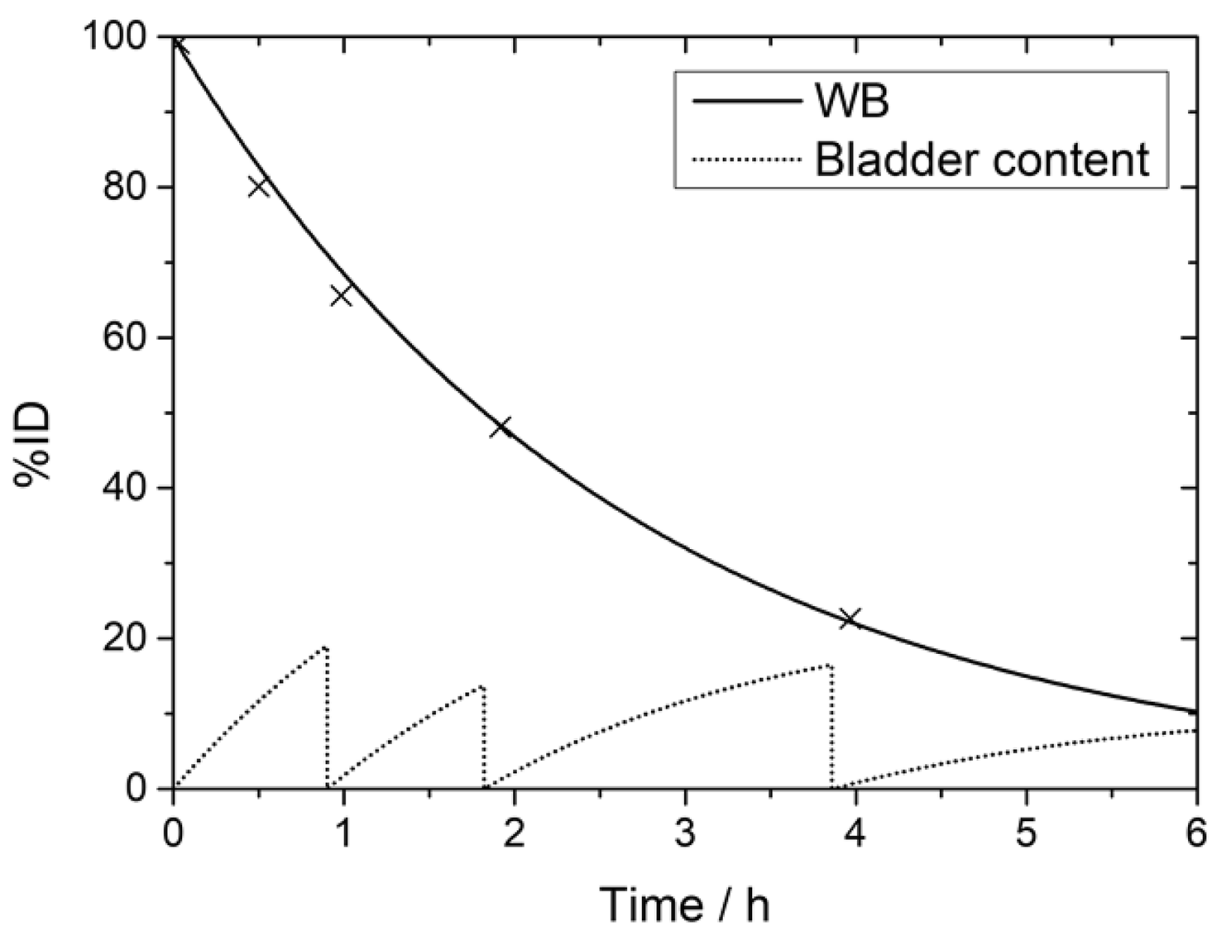
Time-activity curves for Patient 1 (P1) for bladder content and whole body (WB). Micturition prior to imaging at 1 h, 2 h and 4 h after i.v. administration

The biodistribution of [^99m^Tc]Tc-N4-BTG was determined for all major organs in all patients. The patient individual effective half-lives of the tracer are listed in Table 2. Time-integrated-activity curves, expressed as percentage of injected activity (ID), of P1 are shown in Fig. 4 for various organs and in Fig. 5 for bladder content and whole body. Similar time-activity curves were recorded for all four patients. The highest pancreatic uptake was observed in P1 at 1 h p.i. with approximately 1.2% of ID. The data at later time points showed a decrease below 0.2% at 4 h. For all patients, the mean pancreas uptake at 1 h p.i. was (0.73 ± 0.37)% with a fast effective half-life of about 1 h (Table 2). At 1 h p.i., the highest organ uptake (besides the urinary bladder) was observed in the kidneys of P2 with about 2.1% ID, which decreased below 0.3% at 4 h p.i. In all patients, the mean uptake in the kidneys declined from (1.3 ± 0.6)% at 1 h to (0.44 ± 0.13)% at 4 h after injection.

**Table 2:** Determined effective half-lives [h] of [^99m^Tc]Tc-N4-BTG for various organs

Organ	Patient 1	Patient 2	Patient 3	Patient 4	Mean	SD
Heart	1.6	1.0	2.2	1.8	1.7	0.6
Kidneys	2.7	1.1	3.2	3.3	2.6	1.0
Liver	2.8	1.3	2.9	2.3	2.3	0.8
Lungs	1.2	0.79	N/A*	N/A*	1.0	0.3
Pancreas	1.1	0.82	0.79	1.1	0.96	0.18
Red bone marrow	2.7	1.7	6.0	4.4	3.7	1.9
Spleen	2.5	0.93	1.9	2.7	2.0	0.8
WB	1.9	1.8	2.2	2.1	2.0	0.2
WB − Bladder	1.8	1.7	2.1	1.9	1.9	0.2

*Was not assessed as the values did not differ from the background

### Dosimetry

Time-integrated activity coefficients of segmented organs were estimated for each patient individually. Mean values and standard deviations were calculated (Table 3). Highest time integrated activity coefficients were determined in the urinary bladder (0.67 ± 0.02 h), red bone marrow (0.071 ± 0.028 h), kidneys (0.065 ± 0.012 h) and liver (0.063 ± 0.011 h). Mean organ absorbed dose coefficients as well as the effective dose for a standard administered activity of 750 MBq are listed in Table 4.

**Table 3:** Determined time-integrated activity coefficients [MBq-h/MBq] of [^99m^Tc]Tc-N4-BTG for various organs

Organ	Patient 1	Patient 2	Patient 3	Patient 4	Mean	SD
Heart	0.029	0.016	0.019	0.012	0.019	0.007
Kidneys	0.066	0.080	0.060	0.054	0.065	0.012
Liver	0.062	0.056	0.078	0.056	0.063	0.011
Lungs	0.032	0.016	N/A	N/A	0.024	0.012
Pancreas	0.042	0.011	0.021	0.018	0.023	0.014
Red bone marrow	0.068	0.043	0.109	0.063	0.071	0.028
Spleen	0.004	0.005	0.003	0.006	0.005	0.002
Urinary bladder	0.67	0.66	0.66	0.70	0.67	0.02
Remainder of body	2.4	2.3	2.8	2.6	2.5	0.3

**Table 4:** Organ Absorbed Dose Coefficients and Effective Dose of [^99m^Tc]Tc-N4-BTG.

Target Organ	Mean (mGy/MBq)	SD (mGy/MBq)
Adrenals	0.0018	0.0002
Brain	0.0011	0.0002
Breasts	0.0009	0.0001
Gallbladder Wall	0.0018	0.0002
LLI Wall	0.0030	0.0002
Small Intestine	0.0022	0.0002
Stomach Wall	0.0016	0.0002
ULI Wall	0.0020	0.0002
Heart Wall	0.0014	0.0002
Kidneys	0.0034	0.0003
Liver	0.0019	0.0004
Lungs	0.0013	0.0003
Muscle	0.0015	0.0002
Pancreas	0.0043	0.0015
Red Marrow	0.0015	0.0003
Osteogenic Cells	0.0039	0.0005
Skin	0.0009	0.0001
Spleen	0.0016	0.0005
Testes	0.0021	0.0002
Thymus	0.0013	0.0002
Thyroid	0.0013	0.0002
Urinary Bladder Wall	0.0281	0.0008
Total Body	0.0015	0.0002
Effective Dose [mSv/MBq]	0.0027	0.0002
Effective Dose [mSv/750MBq]	2.04	0.14

The highest absorbed dose coefficient was observed in the urinary bladder wall (0.028 ± 0.001 mGy/MBq), followed by the pancreas (0.0043 ± 0.0015 mGy/MBq), osteogenic cells (0.0039 ± 0.0005 mGy/MBq), kidneys (0.0034 ± 0.0003 mGy/MBq), and liver (0.0019 ± 0.0004 mGy/MBq).

**Fig. 6:**
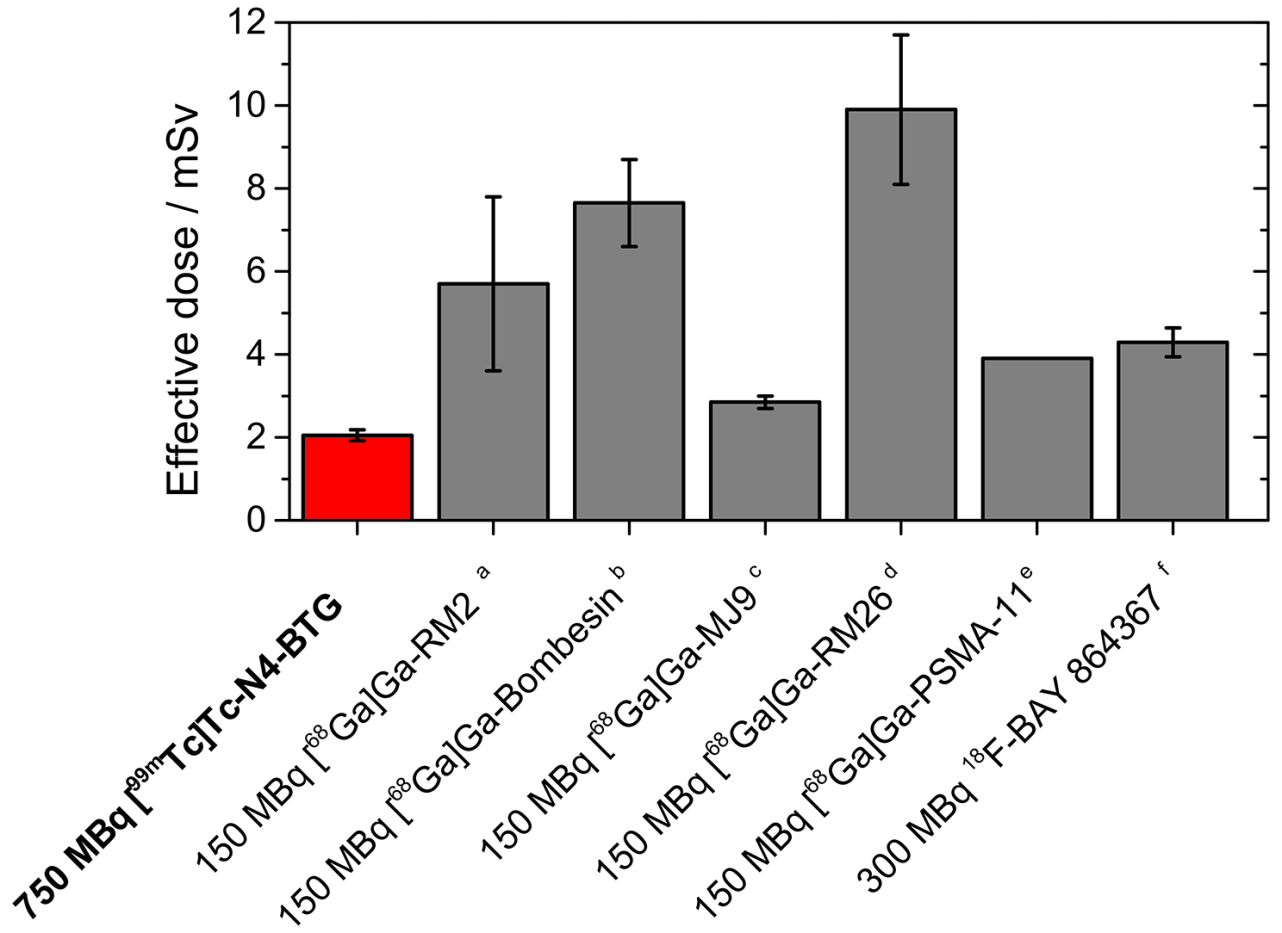
Comparison of effective doses for typical administered activities for various GRPR- and PSMA-targeted compounds. ^a^ [23]; ^b^ [33]; ^c^ [34]; ^d^ [35]; ^e^ [36]; ^f^ [37]

Across the four patients, the effective dose coefficient was 0.0027 ± 0.0002 mSv/MBq, yielding a total effective dose of 2.04 ± 0.14 mSv (ICRP 103) for an injected activity of 750 MBq of [^99m^Tc]Tc-N4-BTG. As additional information, the outdated effective dose using the tissue weighting factors of the ICRP 60 would be 2.46 ± 0.13 mSv. A comparison with published data on other tracers used for the detection of PCa is presented in Fig. 6.

## Discussion

Hybrid imaging with PSMA-directed PET/CT has demonstrated significant advantages in the detection of prostate cancer. However, PSMA expression is observed in only ∼ 90–95% of patients with PCa. As a complementary approach, bombesin-based tracers have shown promising results in lower-grade, well-differentiated tumours [10] but also in advanced and aggressive PCa [10, 11], which no longer show sufficient PSMA expression [22]. However, these tracers have primarily been available as PET vectors, restricting their use to the higher cost and limited availability of PET systems, especially in rural areas.

In contrast, SPECT scanners are more accessible and less expensive, and the isotope technetium-99m used is available worldwide through via ^99^Mo/^99m^Tc generators. This could open up a niche for particularly smaller hospitals that do not have access to PET. Moreover, due to the expected increase in cancer diseases in the next decades and thus, the need of applicable imaging options, these smaller centres must not be left behind, as the big centres alone cannot cover this demand in the future. However, due to the higher image resolution and better quantification obtainable by PET, (pre-) clinical development currently focuses on the development of improved PET probes. In order to improve SPECT imaging and scintigraphy and thus, strengthen smaller clinics in the detection of cancer diseases, novel tracers have to be designed. Very recently, Günther et al. introduced [^99m^Tc]Tc-N4-BTG, a novel pharmacophore-modified GRPR-directed vector for conventional nuclear medicine imaging, which revealed noticeably improved tumour-to-organ ratios in animals when compared to other bombesin derivatives. Based on these promising results, we aimed to translate this compound into four patients for a proof-of-concept evaluation. This study provides the initial clinical results of biodistribution and normal organ radiation absorbed dose analysis with [^99m^Tc]Tc-N4-BTG.

[^99m^Tc]Tc-N4-BTG could be easily labelled manually, resulting in high molar activities of A_m_∼ 100 GBq/µmol and radiochemical yields of greater than 90%. As expected, [^99m^Tc]Tc-N4-BTG was well tolerated by all patients. No acute or subacute adverse events were observed.

[^99m^Tc]Tc-N4-BTG revealed a favourable biodistribution profile in humans, benefiting from its fast activity clearance from non-tumour organs using the kidneys as the main excretion route, which was already observed in preclinical studies [15]. Similar to previously investigated GRPR antagonists, a rapid activity clearance could be observed, resulting in swift blood clearance. Consequently, background accumulation was low, aiding in image interpretation.

Due to tracer concentration in urine, the urinary bladder uptake was high throughout the whole observation period up to 4 h after tracer injection. The peak pancreatic uptake was noted visually 30 min after injection, followed by a rapid decline until 2 h after injection. Noteworthy, pancreatic uptake showed a high variance in the initial four patients examined, likely due to physiological differences in GRPR-receptor expression, as no pancreatic pathology was found in any of the subjects, although fatty infiltration could potentially reduce uptake [23]. Noteworthy, one patient exhibited high tracer retention in the lymphatic tissue of the Waldeyer’s ring, likely due to inflammation, as observed in previous examinations of bombesin-based tracers [23].

No lesions could be detected in either of these four PCa patients despite rising PSA values following prostatectomy up to 2.4 ng/mL. This is likely due to insufficient or absent GRPR expression or the limited resolution obtainable by ^99m^Tc-based SPECT imaging, alternatively a local recurrent tumour manifestation might have been masked by bladder activity. It must be mentioned that neither of these patients revealed any suspicious lesions in [^68^Ga]Ga-PSMA-I&T PET/CT. Therefore, further examinations in these patients are warranted in the future.

In comparison with other imaging probes used for the detection of PCa, [^99m^Tc]Tc-N4-BTG showed a favourable biodistribution and dosimetry in prostate cancer patients. In comparison to PSMA-targeted compounds, no uptake was observed in the salivary glands, which was expected because GRPR ligands are not known to accumulate in these tissues. With an effective dose of 2.04 ± 0.13 mSv for a standard administered activity of 750 MBq, the effective dose of [^99m^Tc]Tc-N4-BTG was found to be comparable with or even lower than that of clinically applied ^68^Ga- or ^18^F-labelled PSMA inhibitors as well as bombesin tracers (Fig. 6). We anticipate that this is due to its fast clearance kinetics already observed in preclinical models [15]. The effective dose could possibly be reduced to even lower levels by increasing the frequency of bladder voiding and the use of diuretics, as demonstrated for [^68^Ga]Ga-PSMA-HBED CC [24]. Based on these preliminary results, we consider [^99m^Tc]Tc-N4-BTG safe for further clinical use.

The major limitation of this study is its small patient cohort of only four PCa patients in whom no tumour lesions could be unveiled. Therefore, no information on the optimal imaging time point regarding tumour-to-organ ratio can be given. Hence, further research is highly warranted to gain insight into the ideal clinical parameters and PSA range to identify tumour manifestations in PSMA-negative PCa with [^99m^Tc]Tc-N4-BTG.

The last imaging procedures started 4 h after injection. One later imaging time point at e.g. 24 h would have been desirable for a better determination of the effective half-lives leading to a more accurate dosimetry, as a time point between three and five times the effective half-life is recommended e.g. by the Committee on Medical Internal Radiation Dose (MIRD) [25] and by Stabin at al [26]. However, imaging was performed for clinical reasons and later imaging was not indicated as no tumour lesions could be detected and excellent background clearance of the tracer could be observed an the earlier time points.

In general, bombesin-based tracers could take a complementary role to PSMA inhibitors for prostate cancer imaging and might also be applicable in the setting of radioguided surgery. Additionally, given the rising interest in PCa theranostics, a significant rise in the number of patients to undergo radioligand therapy is to be anticipated. Given a substantial percentage of up to 5–10% of PSMA-negative PCa [27], the number of subjects eligible for radioligand therapy addressing GRPR might not be negligible. In this vein, recent pre-clinical and clinical studies have reported on the feasibility of GRPR-directed radionuclide therapy with ^177^Lu-labelled receptor antagonists [12, 28]. Given the widespread availability of gamma camera-based imaging in combination with its lower acquisition costs, [^99m^Tc]Tc-N4-BTG might also aid in the selection of potential therapeutic candidates.

Last, imaging (and therapy) of other GRPR-expressing tumour entities including breast cancer and gastrointestinal stromal tumours might further broaden the scope of applications for [^99m^Tc]Tc-N4-BTG beyond prostate cancer [29–32].

## Conclusions

[^99m^Tc]Tc-N4-BTG appears to be a safe diagnostic agent with a favourable biodistribution. It shows favourable imaging characteristics with fast renal clearance and physiological uptake only in the pancreas, which is mostly cleared within the first two hours after injection.

Compared to GRPR-targeted PET tracers, the ^99m^Tc-labelled agent could contribute to a broader application and better availability of this novel approach, especially in smaller clinical centres. Further evaluation of this novel GRPR ligand is required to elucidate its clinical value for the detection or staging of prostate cancer.

## Data Availability

The datasets generated during and/or analysed during the current study are available from the corresponding author on reasonable request.
